# Confirming the efficacy and safety of CDK4/6 inhibitors in the first-line treatment of HR+ advanced breast cancer: a systematic review and meta-analysis

**DOI:** 10.3389/fphar.2024.1369420

**Published:** 2024-08-05

**Authors:** Xin Guan, Mengyuan Li, Xinyue Ji, Yufei Wang, Lei Tian

**Affiliations:** ^1^ School of International Pharmaceutical Business, China Pharmaceutical University, Nanjing, China; ^2^ Center for Pharmacoeconomics and Outcomes Research, China Pharmaceutical University, Nanjing, China

**Keywords:** CDK4/6 inhibitor, hormone-receptor-positive, advanced breast cancer, fractional polynomials models, meta-analysis

## Abstract

**Objective:** Cyclin-dependent kinase (CDK) 4 and 6 inhibitors (abemaciclib, palbociclib and ribociclib) have been recommended in the first-line treatment of hormone receptor-positive (HR+) breast cancer in China. Our study aims to evaluate the efficacy and safety of CDK4/6 inhibitors by processing survival data using fractional polynomial modeling methods.

**Methods:** Phase II or III randomized controlled trials in treatment-naive HR + patients with advanced breast cancer were systematically searched through the preset search strategy. The fractional polynomial (FP) model was used to relax the proportional hazard assumption and obtain time-varying hazard ratio (HR). Progression-free life years (PFLYs) and life years (LYs) were calculated from the area under curve (AUC) of the predicted progression-free survival (PFS) and overall survival (OS) curves to evaluate the long-term efficacy benefit. Odds ratio (OR) of grade≥3 adverse events were analyzed for safety outcomes.

**Results:** 6 randomized controlled trials with 2,638 patients were included. The first-order FP model (*p* = −1) and the first-order FP model (*p* = 1) were used to calculate the time-varying HR of PFS and OS, respectively. Extrapolating to 240 months, abemaciclib obtained a PFS benefit of 3.059 PFLYs and 6.275 LYs by calculating the AUC of the PFS and OS curves. Palbociclib obtained 2.302 PFLYs and 6.351 LYs. Ribociclib obtained 2.636 PFLYs and 6.543 LYs. In terms of safety, the use of CDK4/6 inhibitors resulted in a higher risk of adverse events (OR = 9.84, 95% CI: 8.13–11.95), especially for palbociclib (OR = 14.04, 95% CI: 10.52–18.90).

**Conclusion:** The use of CDK4/6 inhibitors in treatment-naive patients with HR + advanced breast cancer significantly improves survival, but also increases the risk of adverse events. Abemaciclib and ribociclib may be the best options for prolonging PFS and OS in treatment-naïve patients, respectively.

## 1 Introduction

The status of breast cancer receptors can affect the treatment and prognosis of breast cancer, and the expression of some genes will also be related to the prognosis of different subtypes of breast cancer (Dai et al., 2015; Eom et al., 2016). Hormone receptor-positive (HR+) breast cancer is the subtype with highest percentage, and the progression of the disease is believed to be closely related to estrogen. Endocrine therapy based on aromatase inhibitors is considered to be the standard treatment for HR + breast cancer (Cardoso et al., 2017; Turner et al., 2017). However, disease progression after endocrine therapy resistance poses difficulties and challenges for clinical treatment (Osborne and Schiff, 2011; Milani et al., 2014). Cyclin-dependent kinase (CDK) 4/6 inhibitors are believed to induce G1 phase arrest of tumor cells, and thus exhibit antitumor activity ina variety of solid tumors, especially breast cancer (Sherr and Roberts, 1999; Spring et al., 2020). The emergence of CDK 4/6 inhibitors was an important advance in HR + breast cancer therapy (Kwapisz, 2017; Jerzak et al., 2023). The effectiveness of CDK4/6 inhibitors in HR + breast cancer has been validated in clinical trials regardless of whether patients develop resistance to endocrine therapy (Finn et al., 2015; Cristofanilli et al., 2016; Sledge et al., 2017; Hortobagyi et al., 2018; Tripathy et al., 2018; Johnston et al., 2019; Rugo et al., 2019; Zhang et al., 2020; Slamon et al., 2021; Xu et al., 2022). However, it is necessary to make the optimal choice among different intervention regimens in clinical use. However, there is currently a lack of head-to-head clinical trials between different inhibitors, and indirect comparison can be used as an alternative to direct evidence from clinical trials to compare different treatment regimens (Bucher et al., 1997).

The Chinese breast cancer guidelines recommend a combination of CDK4/6 inhibitors and aromatase inhibitors (AI) as the preferred treatment for HR + advanced breast cancer without endocrine therapy, including abemaciclib, palbociclib, ribociclib. Network meta-analysis (NMA) evidence has partially covered CDK4/6 inhibitors in treatment-naïve HR + breast cancer patients, and the results show that adding CDK4/6 inhibitors to endocrine therapy is an effective choice (Liu et al., 2023; Guo et al., 2024; Kappel et al., 2024). But most of the existing literature uses the hazard ratio (HR) reported in clinical trials and analyses based on the constant proportional hazard (PH) assumption. However, the PH assumption in some studies not valid, such as the crossover in the updated overall survival curve of the MONARCH 3 trial, which indicates that the calculation model of non-PH needs to be considered. Therefore, our study used the non-PH model to conduct NMA on the effectiveness of CDK4/6 inhibitors in the first-line treatment of HR + advanced breast cancer, and directly used survival time instead of HR as an efficacy indicator to more intuitively express the results.

## 2 Methods

### 2.1 Literature search strategy and inclusion criteria

As of 24 May 2024, we systematically searched PubMed and Embase for published studies of clinical trials related to comparative drugs according to predetermined search strategies. Search strategies are shown in Supplementary Table S1. Two reviewers (Ji and Wang) conducted literature search and screening. When there is a dispute, the third reviewer (Li) will make the judgment. Included randomized controlled trials were required to meet the following requirements: 1) The patients were diagnosed with advanced HR + breast cancer and had not previously received advanced systemic therapy. 2. The intervention arm was abemaciclib, palbocilib, ribociclib any inhibitor combined with aromatase inhibitors, while the control arm was treated with aromatase inhibitors alone. 3. Progression-free survival (PFS) or overall survival (OS), as well as grade≥3 adverse events were reported, with rereported results using the latest released version. 4. The study design was prospective, phase II or III, randomized controlled trial (RCT). Controlled trials with small available sample size (N ≤ 30) or single-arm trials were excluded, and patients in premenopause or perimenopause who would receive additional ovarian function inhibitors were excluded.

The Preferred Reporting Items for Systematic Reviews and Meta-Analyses (PRISMA) checklist for our study is provided in SupplementaryAppendix S1 (Page et al., 2021).

### 2.2 Risk of bias assessment and data extraction

The Cochrane Bias Risk Assessment Tool was used to assess the risk of bias. Two reviewers (Ji and Wang) assessed the quality of the trial. Detailed clinical trial data were extracted from the reports, including experimental design, subject population, sample size, interventions and the hazard ratio.

### 2.3 Statistical analysis

Specific progression-free life years (PFLYs) and life years (LYs) were used as outcome indicators reflecting patient survival, and PFLYs and LYs were calculated using the area under the curve (AUC) of PFS and OS curve. Survival curves were based on the MONARCH 3 aromatase inhibitor arm, as this trial had a longer follow-up period and both PFS and OS curves were available. The HR obtained from the NMA was used to calculate the survival rates for different protocols. We used GetData 2.26 to digitize the survival curve and used Guyot method to obtain individual patient data from Kaplan-Meier (KM) curve (Guyot et al., 2012). Six conventional standard parameter models (exponential, gamma, gompertz, weibull, log-logistic, log-normal) were considered in the fitting of the survival curve of the aromatase inhibitor arm.

In NMA, methods for comparing survival data are often based on the proportional hazard (PH) assumption. However, the premise of the PH assumption will no longer be satisfied when the special case of intersecting survival curves arises. Jansen proposed a method for NMA on survival data using a fractional polynomial (FP) model, which allows flexible modeling of HR independent of PH assumptions (Jansen, 2011). We used cumulative hazard to confirm the PH assumption, and the log cumulative hazard plots are shown in Supplementary Figure S1. Some clinical trials do not satisfy the PH assumption because the curves intersect or overlap.

The first-order FP model models the survival data of different treatment groups to obtain the HR changing over time. The model can correspond to different first-order FP models by changing the parameter P, and the parameter P is selected from the set: 2, −1, −0.5, 0, 0.5, 1, 2, 3. The calculation of time-varying HR comes from the model parameters of the optimal model. The simplified first-order FP model is calculated by the Formula 1 (Wiksten et al., 2020), where d0 and d1 are the difference between the effect coefficients of protocol A and protocol B, and g_0_(t) and g_1_(t) are functions of time. We used HR to plot survival curves for different treatments and predicted the expected survival curve for each treatment over a 20-year period.

$${\text{HR}}_{\text{AB}}\left(t\right)=e^{\left(g_{0}\left(t\right)*d0+g_{1}\left(t\right)*d1\right)}$$ (1)

For severe adverse events (grade≥3), Odd Ratio (OR) was used to explore the correlation between CDK4/6 inhibitors and toxicity. “gemtc” package of Bayesian network meta-analysis was used for statistical analysis, and 4 Markov chains consisting of 50,000 samples were performed for 100,000 iterations. I2 was used to explore the heterogeneity of the study, and random effects model will be used when I2≥40%. The acquisition of time-varying HR and the statistical analysis of safety were achieved by R 4.2.3 and R studio.

## 3 Results

### 3.1 Literature search and study characteristics

**FIGURE 1:**
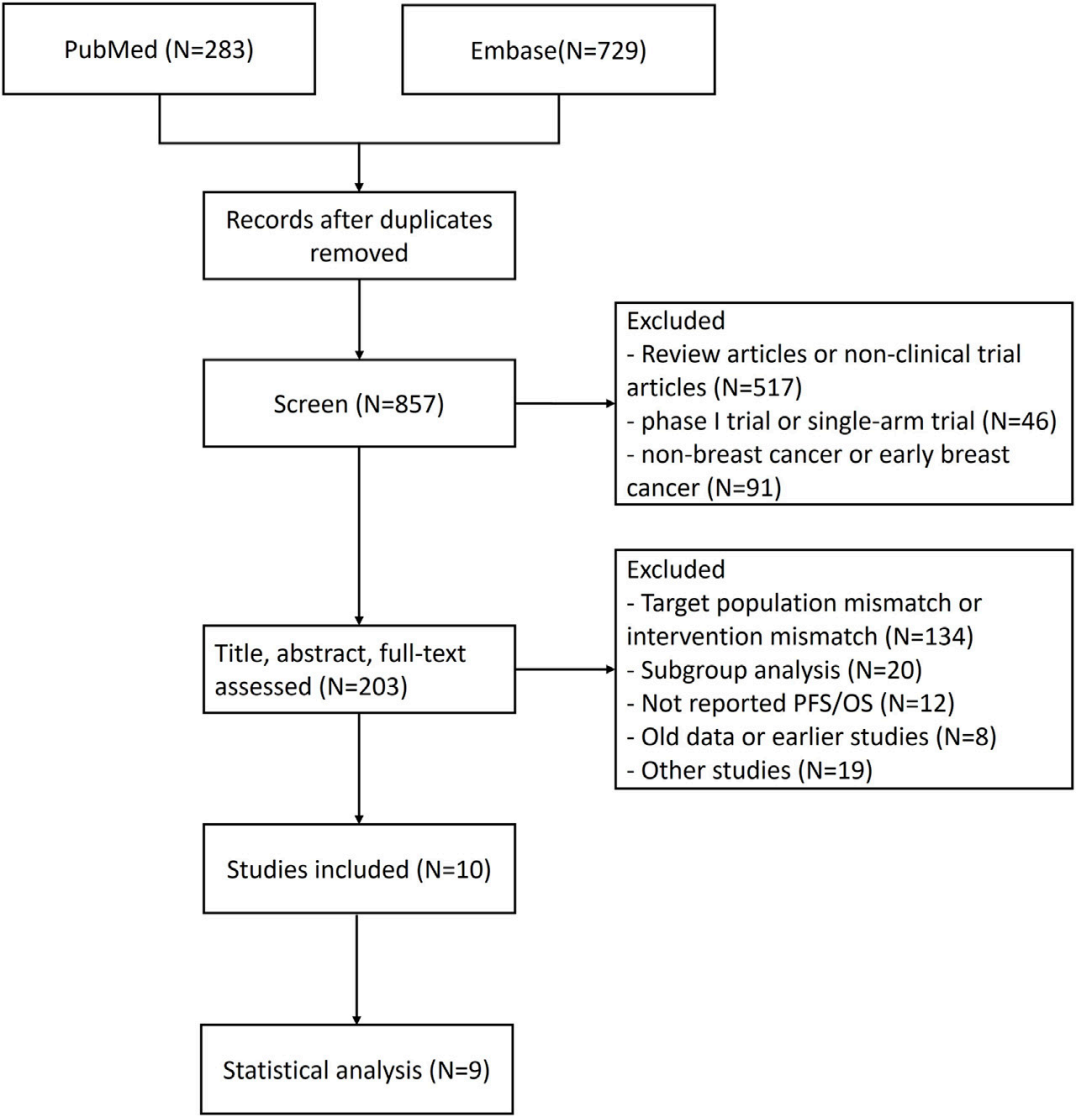
The flow diagram for the selection of articles and studies.

**FIGURE 2:**
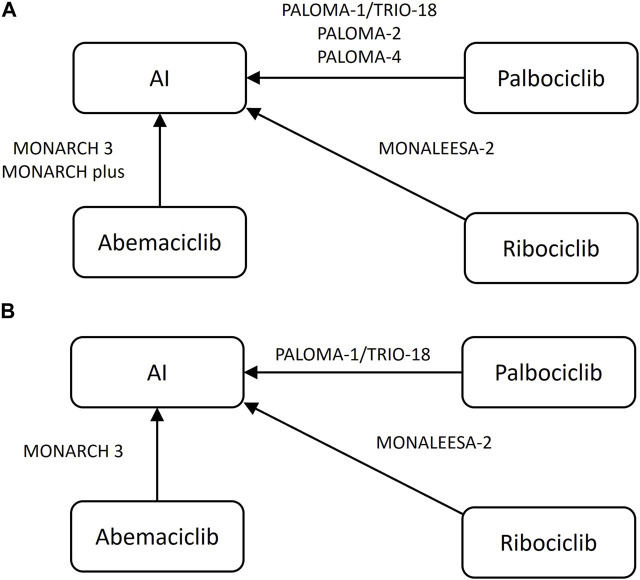
Network plot of included randomized controlled trials **(A)** progression-free-survival, **(B)** overall survival AI, aromatase inhibitor, letrozole or anastrozole.

1,012 study records were identified through the search strategy and 155 duplicate records were removed. Firstly, 654 irrelevant study records were preliminarily excluded through terms such as “meta-analysis,” “single-arm” and “neoadjuvant/adjuvant therapy.” After evaluating the title, abstract, and full-text, 193 records were deleted according to the criteria. The specific flow diagram of our NMA is shown in Figures 1, 2. A total of nine research articles and one conference abstract were retrieved, corresponding to 6 RCTS, for ribociclib, abemacicilib, palbociclib combined with letrozole/anastrozole (Finn et al., 2015; Hortobagyi et al., 2018; Johnston et al., 2019; Rugo et al., 2019; Finn et al., 2020; Zhang et al., 2020; Finn et al., 2022; Hortobagyi et al., 2022; Xu et al., 2022; Goetz et al., 2024). The key information of RCTS is shown in Table 1, and some characteristics of enrolled patients were shown in Supplementary Table S2. But there were no reported KM curves in the conference abstracts (Finn et al., 2022), so it was not included in the statistical analysis.

**TABLE 1:** The characteristics of trials included in network meta-analysis.

Study	NCT	Design	ARM 1	ARM 2	PFS		OS		Any AEs, grade≥3 n (%)	
					Median follow-up, months	HR (95% CI)	Median follow-up, months	HR (95% CI)	ARM 1	ARM 2
MONALEESA-2 (Hortobagyi et al., 2018; Hortobagyi et al., 2022)	NCT01958021	Phase III, randomized, double-blind	ribociclib + letrozole	Placebo + letrozole	26.4	0.568 (0.457–0.704)	79.2	0.76 (0.63–0.93)	297 (89%)	140 (42%)
MONARCH 3 (Johnston et al., 2019; Goetz et al., 2024)	NCT02246621	Phase III, randomized, double-blind	abemaciclib + letrozole/anastrozole	Placebo + letrozole/anastrozole	26.7	0.540 (0.418–0.698)	97.2	0.804 (0.637–1.015)	227 (69%)	46 (29%)
MONARCH plus (Zhang et al., 2020)	NCT02763566	Phase III, randomized, double-blind	abemaciclib + letrozole/anastrozole	Placebo + letrozole/anastrozole	16.0	0.499 (0.346–0.719)	NR	NR	121 (59%)	23 (23%)
PALOMA-1/TRIO-18 (Finn et al., 2015; Finn et al., 2020)	NCT00721409	Phase II, randomized, open-label	palbociclib + letrozole	Placebo + letrozole	29.6	0.488 (0.319–0.748)	64.7	0.897 (0.623–1.294)	63 (76%)	16 (21%)
PALOMA-2 (Rugo et al., 2019; Finn et al., 2022)	NCT01740427	Phase III, randomized, double-blind	palbociclib + letrozole	Placebo + letrozole	37.6	0.563 (0.461–0.687)	90.0	0.956 (0.777–1.177)^a^	364 (82%)	67 (30%)
PALOMA-4 (Xu et al., 2022)	NCT02297438	Phase III, randomized, double-blind	palbociclib + letrozole	Placebo + letrozole	52.8	0.677 (0.529–0.867)	NR	NR	149 (88%)	36 (21%)

^a^ There is no Kaplan-Meier curve of overall survival, which is not included in statistical analysis.

HR+, hormone receptor-positive; HER2-, human epidermal growth factor receptor 2-negetive; PFS, progression-free survival; OS, overall survival; HR, hazard ratio; NR, not reported; AEs, adverse events.

The results of bias analysis are presented in Supplementary Figures S2, S3. For all RCTs, the risk of bias was generally low. Only PALOMA-1 used an open-label design and is considered high risk on blinding of participants and personnel. The MONARCH 3 and PALOMA-2 trials reported results from independent central review and were considered low risk on blinding of outcome assessment, while the remaining trials were considered unclear risk of bias.

### 3.2 Efficacy outcomes

**FIGURE 3:**
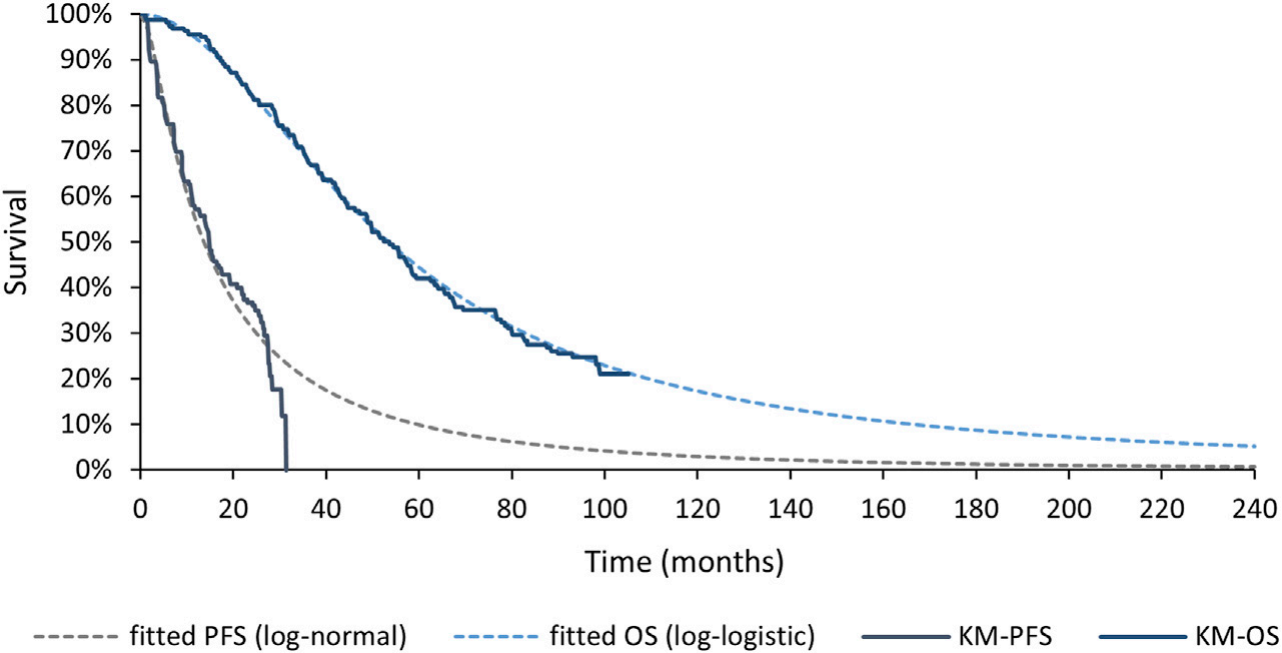
Fitted Survival Curves for PFS and OS of MONARCH 3 aromatase inhibitor arm. PFS, progression-free survival; OS, overall survival; KM, Kaplan-Meier.

Log-normal model was applied to PFS curve of MONARCH 3 aromatase inhibitor arm and log-logistic model was applied to OS curve. Details of the processing process are shown in the Supplementary Table S3; Supplementary Figure S4. Both goodness of fit and the coincidence degree of curves indicated the rationality of the fitting model selection. The survival curve after fitting is shown in Figure 3.

HR was analyzed using first-order fractional polynomial model, and Table 2 gives information about the different first-order FP models, including AIC values. However, NICE DSU 21 suggests that a minimum AIC may be help in selecting a model that fits the data within the length of follow-up, but the AIC provides little information about how well the model extrapolates to longer time points (Rutherford et al., 2020). When fitting the immature survival data, the fitting model with the minimum value of AIC may have the problem of over-fitting to the tail data. Therefore, we select the best model through Akaike Information Criteria (AIC) and visual inspection.

**TABLE 2:** AIC for all first-order fractional polynomial models.

Parameter	g_0_(t)	g_1_(t)	PFS	OS
−2	1	t^-2^	2,719.43	2,461.94
−1	**1**	t^-1^	**2,559.76**	2,336.31
−0.5	1	t^-0.5^	2,580.92	2,204.08
0	1	log(t)	2,655.93	2,155.94
0.5	1	t^0.5^	2,702.82	2,128.82
1	1	t	2,753.59	**2,060.18**
2	1	t^2^	2,840.56	2,286.93
3	1	t^3^	2,950.63	2,382.09

Bold value means the minimum AIC value.

**FIGURE 4:**
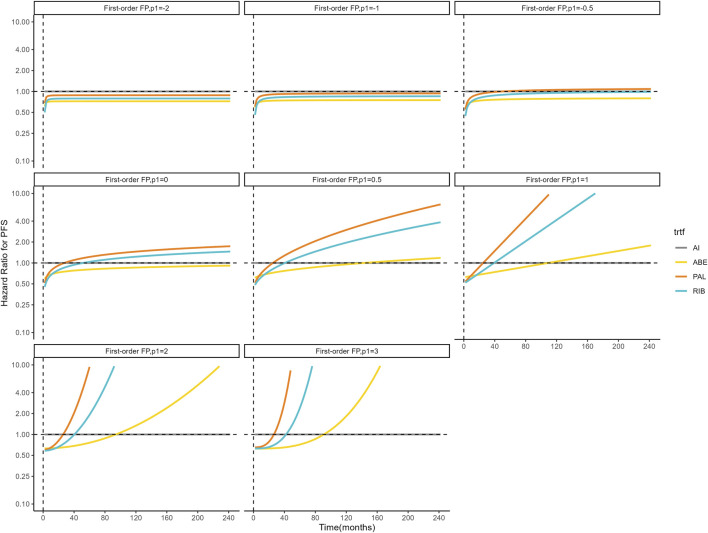
Hazard ratios for PFS (other treatments vs. AI) FP, fractional polynomial; RIB, ribociclib; ABE, abemaciclib; PAL, palbociclib; AI, aromatase inhibitor.

**FIGURE 5:**
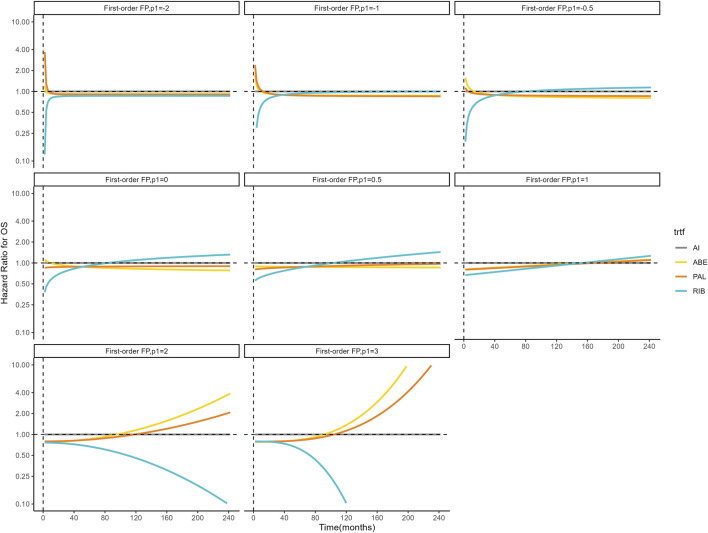
Hazard ratios for OS (other treatments vs. AI) FP, fractional polynomial; RIB, ribociclib; ABE, abemaciclib; PAL, palbociclib; AI, aromatase inhibitor.

**FIGURE 6:**
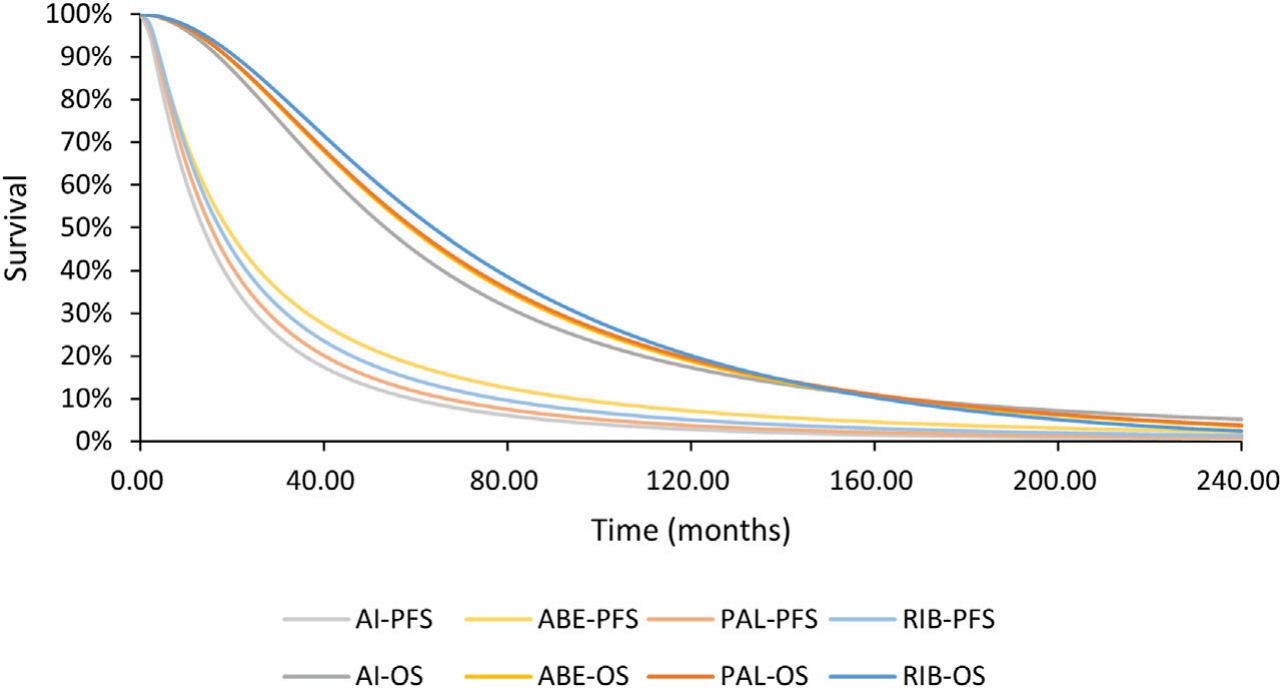
PFS and OS curves for all arms AI, aromatase inhibitor; ABE, abemaciclib; PAL, palbociclib; RIB, ribociclib; PFS, progression-free survival; OS, overall survival.

**FIGURE 7:**
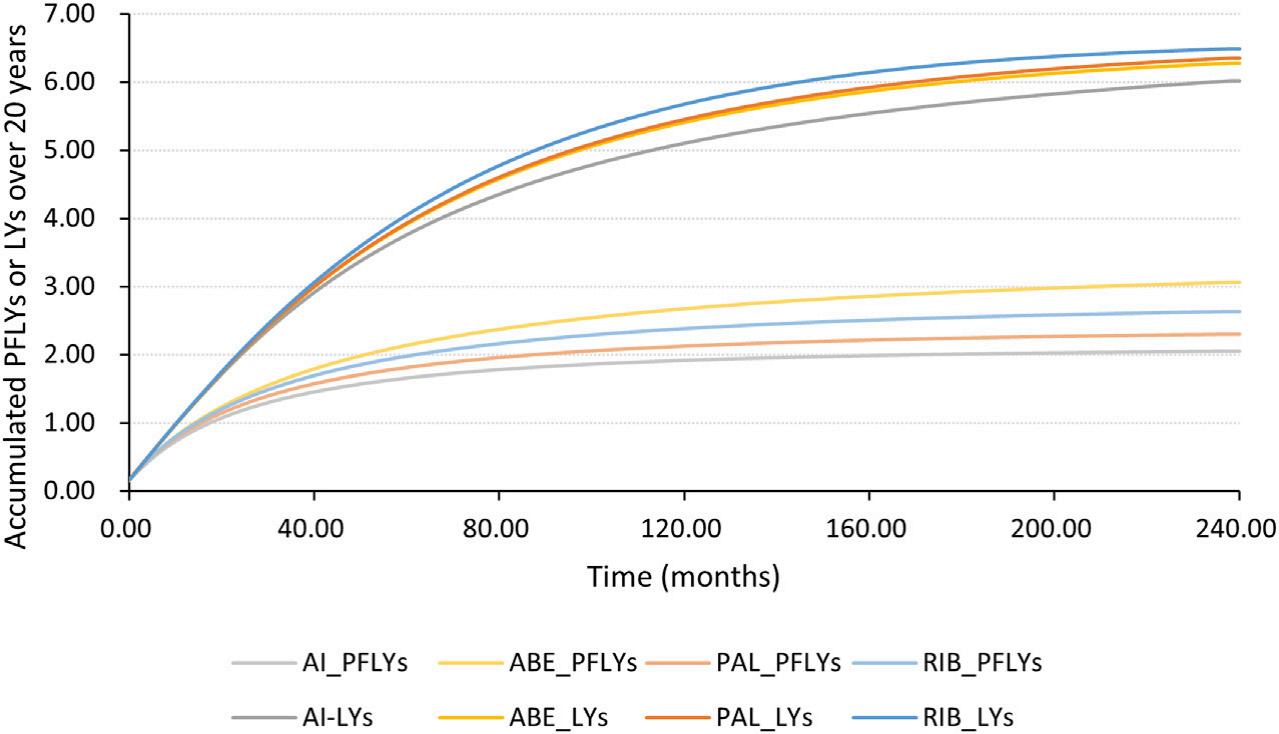
PFLYs and LYs calculated by the AUC of the survival curve, accumulated over 20 years AI, aromatase inhibitor; ABE, abemaciclib; PAL, palbociclib; RIB, ribociclib; PFLYs, progression-free life years; LYs, life years.

Figures 4, 5 shows the time-varying HR for PFS and OS, calculated by all first-order FP models. Some models appear distorted in the long term, that is, HR value was seriously deviated from the clinical practice in long-term, which may due to over-fitting caused by patient detachment and sparse tail data in the late period of the trial. Through the AIC value and visual inspection, we believed that the first-order FP model (*p* = −1) was the optimal time-varying HR calculation model that we should choose in the PFS analysis and the first-order FP model (*p* = 1) was used for OS analysis. Table 3 shows the d0 and d1 required for time-varying HR calculation under the optimal model. By calculating the AUC of the PFS curve, the abemaciclib arm obtained a benefit of 3.059 PFLYs. It was followed by ribociclib arm (2.636 PFLYs), palbociclib arm (2.302 PFLYs) and AI arm (2.047 PFLYs). By calculating the AUC of the OS curve, the ribociclib arm obtained a benefit of 6.543 LYs, followed by palbociclib arm (6.351 LYs), abemaciclib arm (6.275 LYs) and AI arm (6.016 LYs). In Figure 6, the survival curves of abemaciclib, palbociclib, and ribociclib are drawn according to time-varying HR. Figure 7 shows the cumulative PFLYs and LYs calculated over 20 years.

**TABLE 3:** Parameter for HR calculation under the optimal fractional polynomial model.

Group	Parameter	PFS, mean (95% CI)	OS, mean (95% CI)
Abemaciclib vs. AI	d0	−0.286 (−0.494, −0.078)	−0.208 (−0.620, 0.203)
	d1	−0.485 (−1.604, 0.635)	0.001 (−0.005, 0.008)
Palbociclib vs. AI	d0	−0.062 (−0.284, 0.161)	−0.225 (−0.609, 0.158)
	d1	−0.877 (−2.078, 0.323)	0.001 (−0.006, 0.008)
Ribociclib vs. AI	d0	−0.155 (−0.333, 0.023)	−0.407 (−1.027, 0.214)
	d1	−1.243 (−2.345, −0.141)	0.003 (−0.012, 0.018)

AI, aromatase inhibitor.

### 3.3 Safety outcomes

**FIGURE 8:**
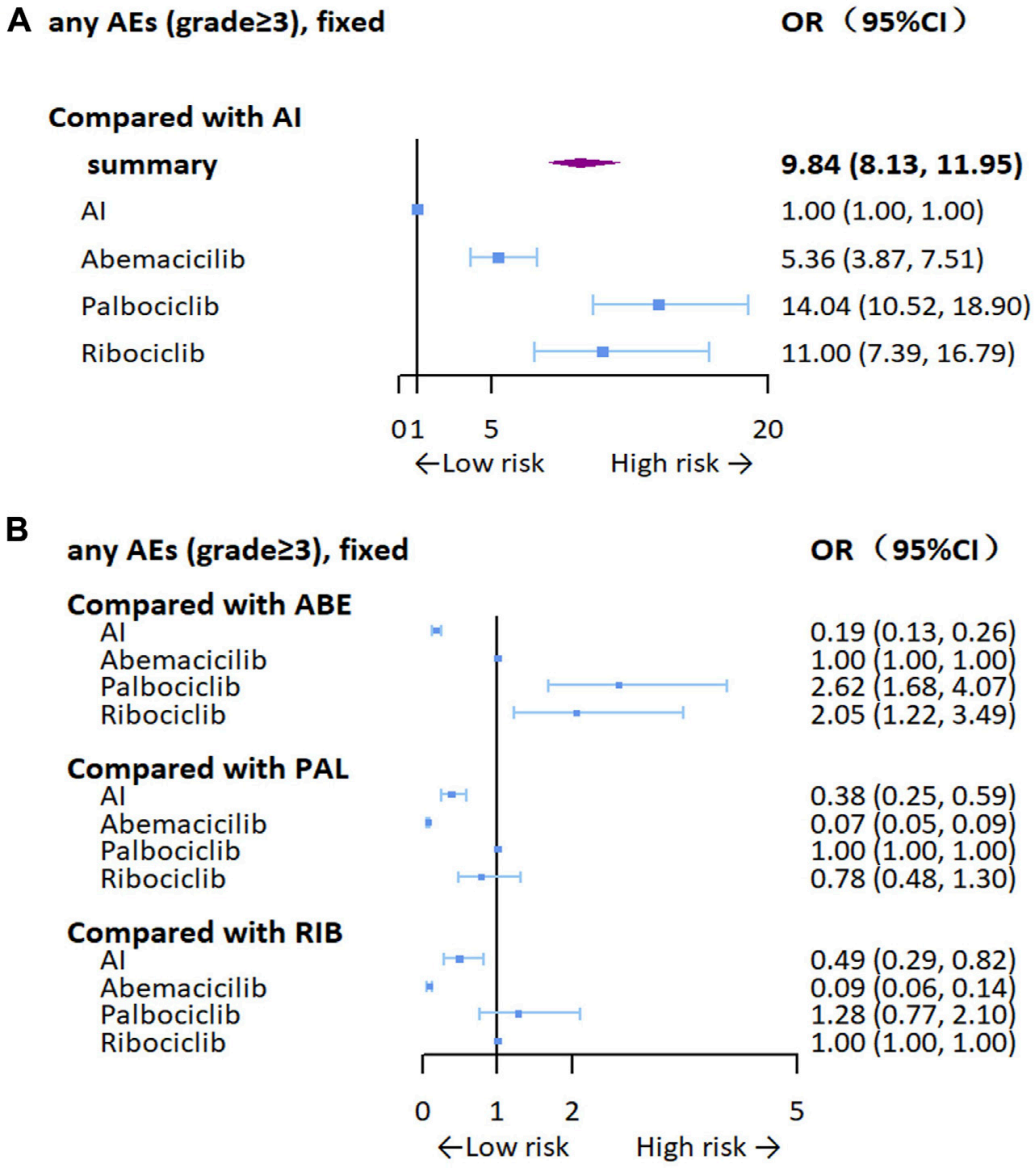
Forest plots for any grade≥3 adverse events. **(A)** Compared with AI, **(B)** compared with the inhibitors AI, aromatase inhibitor; ABE, abemaciclib; PAL, palbociclib; RIB, ribociclib.

We analyzed OR of any grade ≥3 adverse events. As can be seen from Figure 8, the addition of CDK4/6 inhibitors may result in a higher risk of adverse events (OR = 9.84, 95% CI: 8.13–11.95), especially for palbociclib (OR = 14.04, 95% CI: 10.52–18.90), but there was no statistically significant difference in toxicity between ribociclib and palbociclib. We also analyzed clinically common adverse events (grade≥3), including neutropenia, leukopenia, diarrhea, anemia, and ALT/AST increase. In terms of specific adverse event analysis, CDK4/6 inhibitors were significantly associated with an increased risk of adverse events, especially in neutropenia (OR = 105.53, 95% CI: 65.24–183.09) and leukopenia (OR = 46.25, 95% CI: 23.20–110.94), which may be related to the high hematological toxicity of palbociclib and ribociclib. Details of the aggregated and grouped results for the safety outcomes are shown in Supplementary Figure S5.

## 4 Discussion

HR + breast cancer is associated with CDK4/6 activity (Pardee, 1989). CDK 4/6 inhibitors can target the regulation of the cell cycle and can overcome the drug resistance problems associated with endocrine therapy, providing a new pathway for HR + breast cancer treatment (Scott et al., 2017). Since large Phase III trials targeted on abemaciclib, palbociclib and ribociclib, CDK4/6 inhibitors are currently considered the best option for treatment-naive patients with HR + advanced breast cancer. Unfortunately, these three drugs are not directly compared with each other, in which case the network meta-analysis can be a good solution to identify the efficacy of different inhibitors (Kiefer et al., 2015).

We preset inclusion and exclusion criteria for clinical trials and conducted a comprehensive search. 6 eligible RCTs were included in the statistical analysis. The results suggest that the use of CDK4/6 inhibitors is an effective option to prolong survival in patients with both PFLYs and LYs. In terms of predicted survival time, abemaciclib combined AI may be the most effective regimen for prolonging PFS in first-line theatment, while ribociclib combined AI in prolonging OS. However, the use of CDK4/6 inhibitors was associated with an increased risk of adverse events, especially when using palbociclib. We analyzed some common adverse events in clinical, and the use of CDK4/6 inhibitors was also associated with a higher incidence of certain adverse events, particularly neutropenia and leukopenia. But some adverse events were excluded from our analysis, such as QTcF interval prolongation and thromboembolic events, because of limited data reported from clinical trials.

The efficacy of different combinations of CDK4/6 inhibitors in the treatment of breast cancer was not only validated by trials, but also confirmed by meta-analysis. Messina et al. found that the addition of CDK4/6 inhibitors could significantly improve PFS, but it would lead to a significant increase in the occurrence of severe adverse events (Messina et al., 2018). Onesti and Jerusalem (2021) further determined that palbociclib and ribociclib were associated with high hematological toxicity, which was basically consistent with our study results, and abemaciclib was associated with gastrointestinal toxicity (grade1-2 diarrhea), whereas only grade≥3 diarrhea was included in our study. Schettini et al. (2020) mainly analyzed the benefits of CDK4/6 inhibitors on overall survival, which made up for the shortcomings of previous meta-analysis in overall survival analysis. Liu et al. (2023) ranked different combinations of CDK4/6 inhibitors and endocrine therapy according to cumulative probability, and the results showed that palbociclib combined with fulvestrant and abemaciclib combined with fulvestrant may be effective programs in improving PFS and OS, respectively. However, this study did not make clear whether the patients were first-line treatment, which would affect the applicability of the results in the treatment-naïve patients to a certain extent (Liu et al., 2023). Guo et al. (2024) conducted a Bayesian NMA on RCTs with CDK4/6 inhibitors in first-line treatment, and the results showed that ribociclib plus fulvestrant may be the best choice for first-line treatment in terms of improving PFS and OS, but this use has not been approved in China.

The implementation of NMA is based on the assumption of consistency and transitivity. When the research output takes HR as an indicator, it needs to satisfy the constant hazard ratio, that is, HR does not change over a certain period of time. When the proportional hazard assumption is violated within the trial, survival data can be significantly biased if interventions are evaluated using a PH model (Jansen, 2011).

In our study, based on the results of validation of the PH assumption of the clinical trial, some included trials did not conform to the PH assumption. The development of NMA should consider the non-PH model. The FP model was used to relax PH assumption and obtain HR values over time, and to calculate predicted long-term PFLYs and LYs to measure efficacy, validate the efficacy of CDK4/6 inhibitors in treatment-naïve patients, and distinguish the effects of different inhibitors.

However, it is important to note that there were differences in the baseline characteristics of patients included in our study, particularly the MONARCH plus and PALOMA-4 studies, which were focused on Asian populations. As shown in Supplementary Table S2, patients in these two studies were significantly younger, which may be due to the lower age of breast cancer diagnosis in China (Fan et al., 2014). In our study, we used individual patient data from survival curve reconstruction, so we could not adjust for patient baseline characteristics, and differences in patient baseline characteristics introduced potential bias into the results. Second, our study included only six randomized controlled trials of first-line therapy, which may limit the applicability of our results outside the target population to some extent and hinder the development of subgroup analyses due to the small number of trials. In addition, it must be noted that due to the reduction of sample size and sparse data during the later follow-up period, as shown in Figures 4, 5, the long-term HR values predicted by some models are significantly different from the actual situation in some FP model. Although the FP model can simulate HR flexibly, it is affected by the small sample size in the prediction, which increases the uncertainty of our study.

## 5 Conclusion

Our findings confirm that adding CDK4/6 inhibitors to the regimen of treatment-naïve HR + patients with advanced breast cancer significantly improves survival. From the predicted survival curve, abemaciclib may be the most effective regimen for prolonging PFS in first-line therapy, and ribociclib for prolonging OS. In terms of safety, the use of CDK4/6 inhibitors may result in an increased risk of adverse events and should be noted when used clinically, especially the hematological toxicity of palbociclib and ribociclib. However, the evidence from NMA is of limited validity, and more relevant clinical trials or direct evidence from head-to-head clinical trials are expected in the future.

## Data Availability

The original contributions presented in the study are included in the article/Supplementary Material, further inquiries can be directed to the corresponding author.
