# Supplementary material for: Confirming the efficacy and safety of CDK4/6 inhibitors in the first-line treatment of HR+ advanced breast cancer: a systematic review and meta-analysis
Source: Front Pharmacol. 2024 Aug 5;15:1369420. doi: 10.3389/fphar.2024.1369420 (PMC11330780; doi:10.3389/fphar.2024.1369420)
Supplement: Supplementary file 2 [file DataSheet1.docx]

Supplementary Material

Confirming the Efficacy and Safety of CDK4/6 Inhibitors in the First-line Treatment of HR+ Advanced Breast Cancer: A Systematic Review and Meta-Analysis

# Supplementary Table S1. Search Strategies

**PubMed**

|  | Items |
| --- | --- |
| #9 | #8 AND (clinical trial[Filter] OR randomized controlled trial[Filter]) |
| #8 | #3 AND #6 AND #7 |
| #7 | #4 OR #5 |
| #6 | #1 OR #2 |
| #5 | (((abemaciclib[Title/Abstract]) OR (palbociclib[Title/Abstract])) OR (ribociclib[Title/Abstract])) |
| #4 | "cyclin dependent kinase inhibitor"[Title/Abstract] OR "cdk inhibitor"[Title/Abstract] OR "cyclin dependent kinase 4"[Title/Abstract] OR "cyclin dependent kinase 6"[Title/Abstract] |
| #3 | (((cyclin dependent kinase inhibitor[Title/Abstract]) OR (CDK inhibitor[Title/Abstract])) OR (cyclin dependent kinase 4[Title/Abstract])) OR (cyclin dependent kinase 6[Title/Abstract]) |
| #2 | (((advanced breast cancer[MeSH Terms]) OR (advanced breast carcinoma[MeSH Terms])) OR (breast tumor[MeSH Terms])) OR (mammary cancer[MeSH Terms]) |
| #1 | ((((breast cancer[MeSH Terms])) OR (breast carcinoma[MeSH Terms])) OR (breast tumor[MeSH Terms])) OR (mammary cancer[MeSH Terms]) |

**Embase**

|  | Items |
| --- | --- |
| #10 | #6 AND #8 AND #9 |
| #9 | #3 AND #7 |
| #8 | #4 OR #5 |
| #7 | #1 OR #2 |
| #6 | 'randomized controlled trial'/exp OR 'randomized controlled study' OR 'randomized controlled trial' |
| #5 | 'abemaciclib'/exp OR 'abemaciclib' OR 'verzenio' OR 'palbociclib'/exp OR 'ibrance' OR 'palbociclib' OR 'ribociclib'/exp OR 'kisqali' OR 'ribociclib' |
| #4 | 'cyclin dependent kinase inhibitor'/exp OR 'cyclin dependent kinase inhibitor' OR 'cyclin dependent protein kinase inhibitor' OR 'cyclin dependent kinase 4'/exp OR 'cyclin dependent kinase 4' OR 'cyclin dependent protein kinase 4' OR 'cyclin dependent kinase 6'/exp OR 'cyclin dependent kinase 6' OR 'cyclin dependent protein kinase 6' |
| #3 | 'hormone receptor positive' OR 'oestrogen receptor positive' OR 'progesterone receptor positive' OR 'hr positive' OR 'er positive' OR 'pr positive' |
| #2 | 'advanced breast cancer'/exp OR 'advanced breast cancer' OR 'advanced breast carcinoma' OR 'advanced mammary cancer' OR 'advanced mammary carcinoma' |
| #1 | 'breast cancer'/exp OR 'breast cancer' OR 'breast gland cancer' OR 'breast gland neoplasm' OR 'breast malignancies' OR 'breast malignancy' OR 'breast tumor malignant' OR 'mamma cancer' OR 'mammary cancer' OR 'mammary gland cancer' OR 'mammary gland malignancy' OR 'mammary malignancies' OR 'mammary malignancy' |

# Supplementary Table S2. Basic characteristics of patients in included studies

| Study | NCT | Region | Patient | Line | Treatment | | Sample Size | | Age  (median, years) | | ECOG PS (0,≥1) | |
| --- | --- | --- | --- | --- | --- | --- | --- | --- | --- | --- | --- | --- |
|  |  |  |  |  | ARM 1 | ARM 2 | ARM 1 | ARM 2 | ARM 1 | ARM 2 | ARM 1 | ARM 2 |
| MONALEESA-2 | NCT01958021 | global | postmenopausal women with HR+, HER2-recurrent/ metastatic breast cancer | 1st | Ribociclib + letrozole | Placebo + letrozole | 334 | 334 | 62 | 63 | 205/129 | 202/132 |
| MONARCH 3 | NCT02246621 | global | patients with HR+, HER2- advanced breast cancer | 1st | Abemaciclib + letrozole/anastrozole | Placebo + letrozole/anastrozole | 328 | 165 | 63 | 63 | 192/136 | 104/61 |
| MONARCH plus | NCT02763566 | asia | postmenopausal female patients with HR+, HER2- locoregionally recurrent or metastatic breast cancer | 1st | Abemaciclib + letrozole/anastrozole | Placebo + letrozole/anastrozole | 207 | 99 | 54 | 54 | NR | NR |
| PALOMA-1 /TRIO-18 | NCT00721409 | global | postmenopausal women with advanced estrogen receptor-positive and HER2-negative breast cancer | 1st | Palbociclib + letrozole | Placebo + letrozole | 84 | 81 | 63 | 64 | 46/38 | 45/36 |
| PALOMA-2 | NCT01740427 | global | women with estrogen receptor-positive / HER2- advanced breast cancer | 1st | Palbociclib + letrozole | Placebo + letrozole | 444 | 222 | 62 | 61 | 257/187 | 102/119 |
| PALOMA-4 | NCT02297438 | asia | postmenopausal women with a diagnosis of estrogen receptor-positive advanced breast cancer | 1st | Palbociclib + letrozole | Placebo + letrozole | 169 | 171 | 54 | 54 | 84/85 | 81/90 |

# Supplementary Figure S1. Log Cumulative Hazard Plots for Survival Curves

| 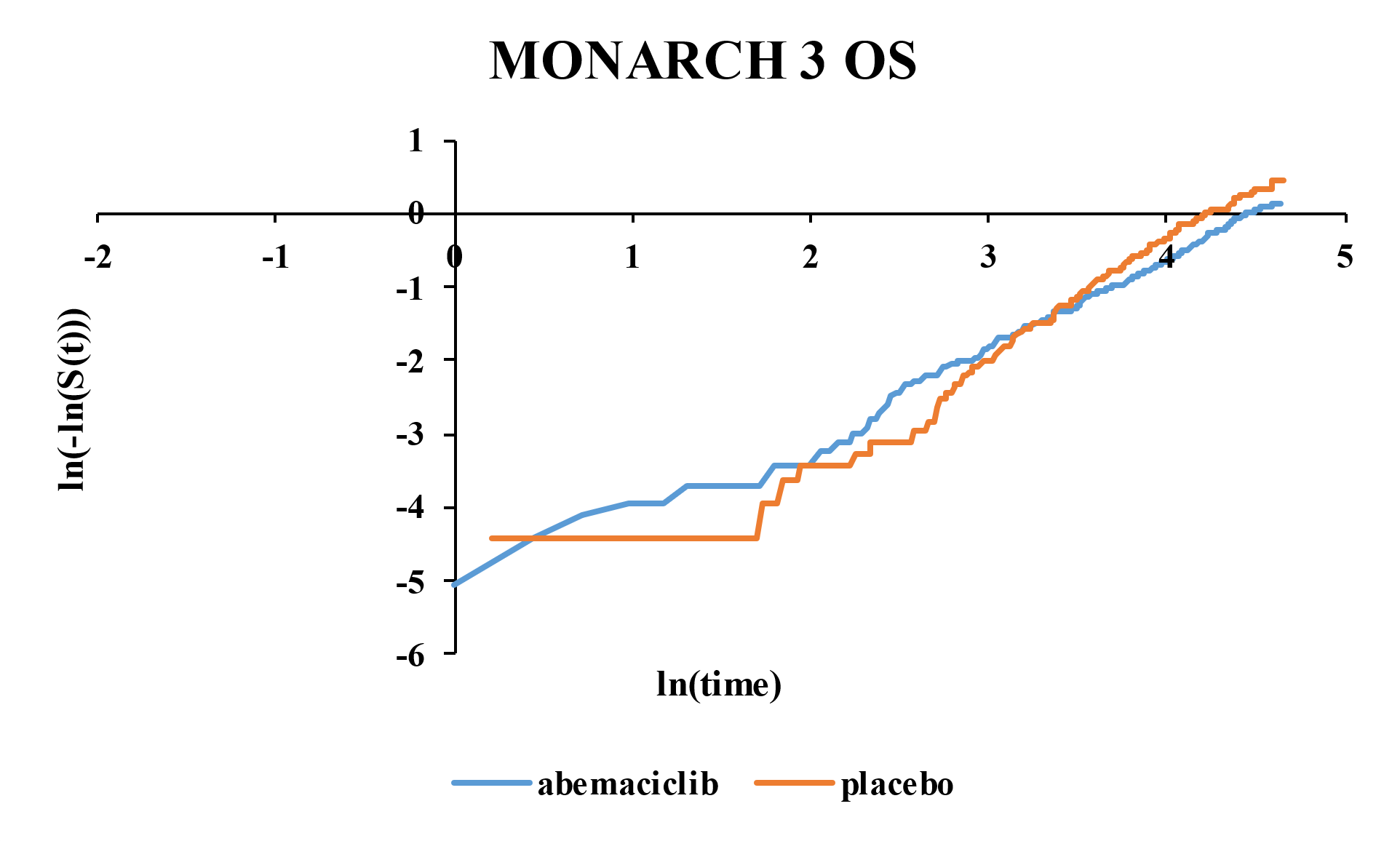 | 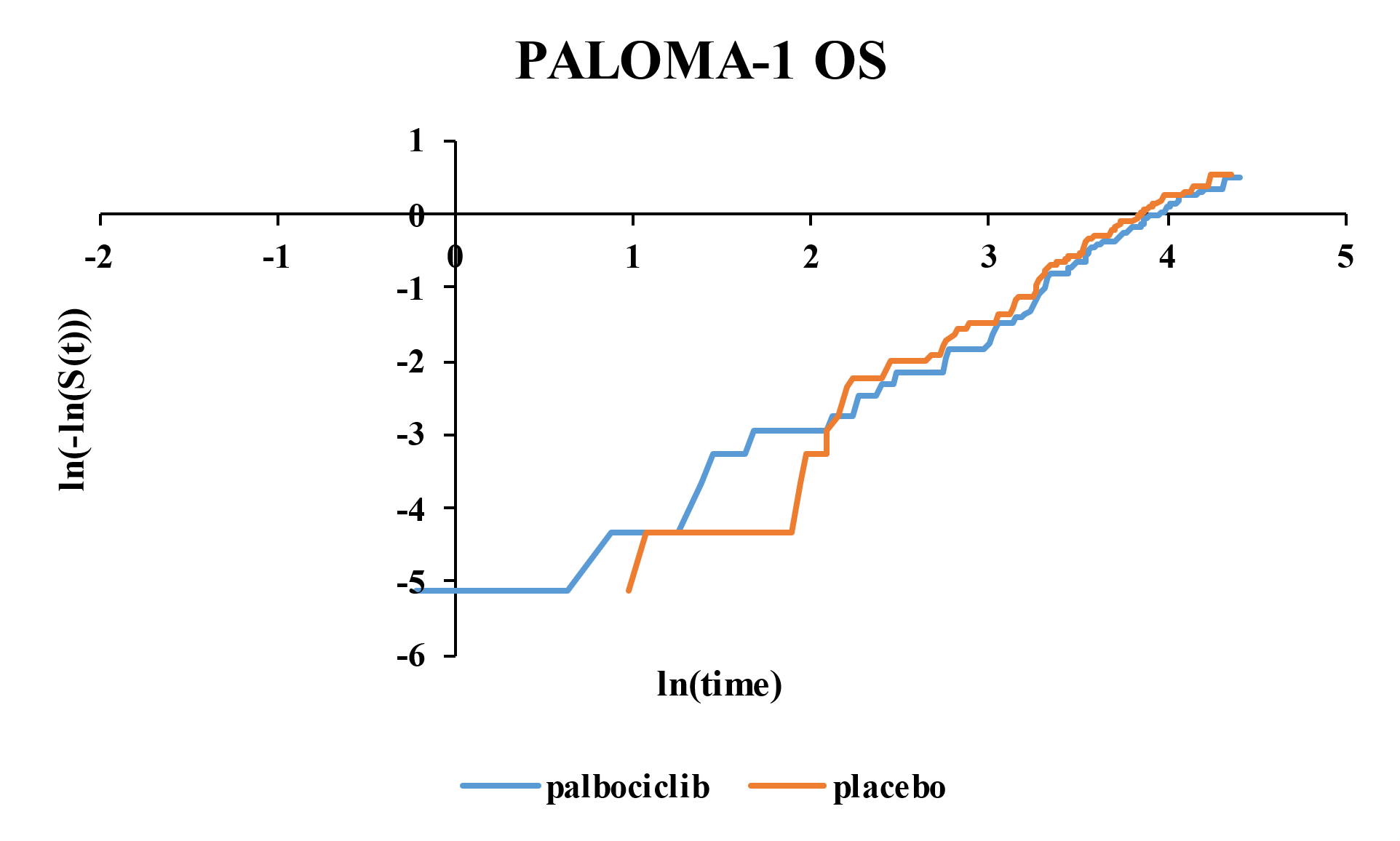 |
| --- | --- |
| 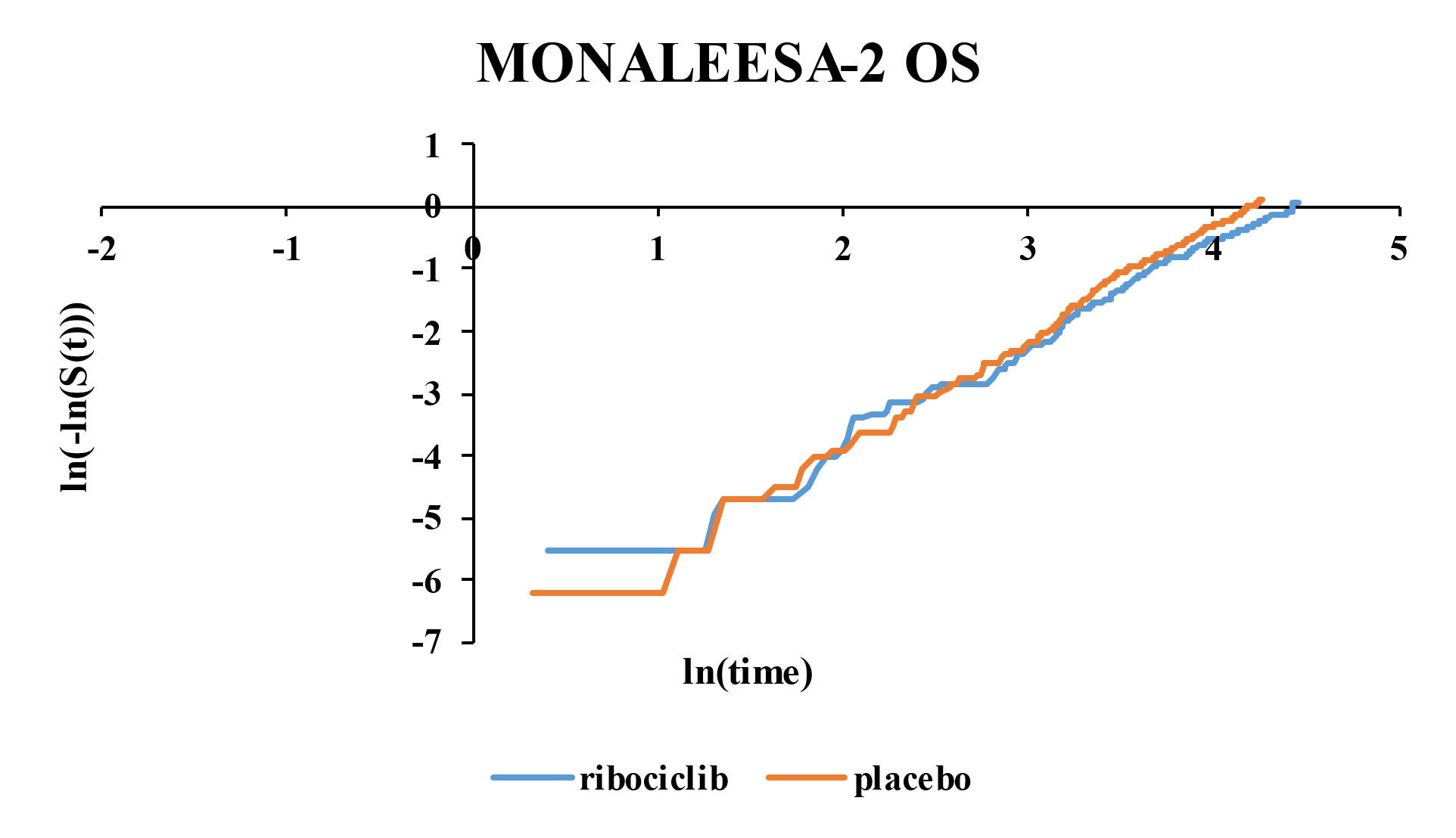 |  |
| 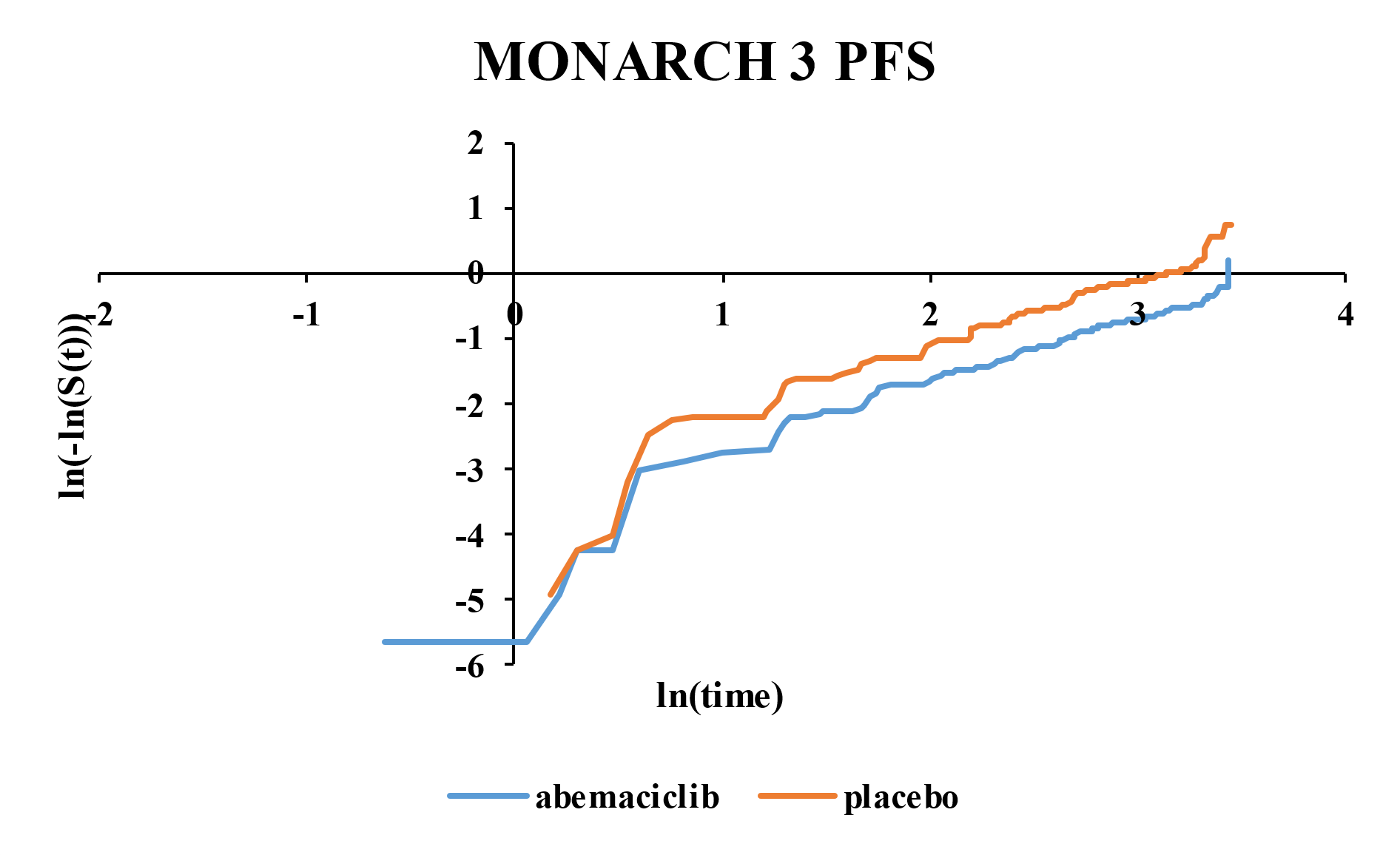 | 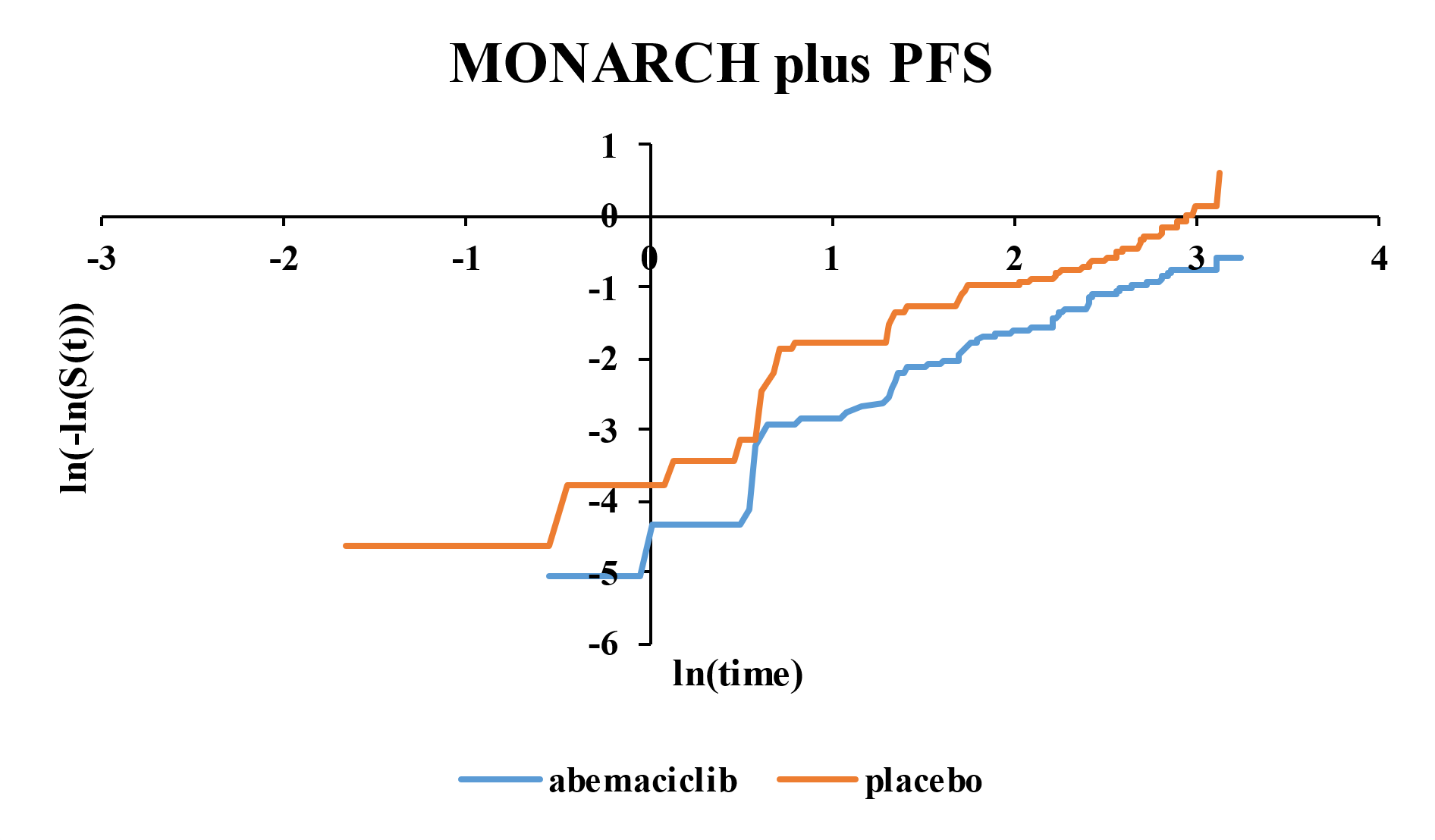 |
| 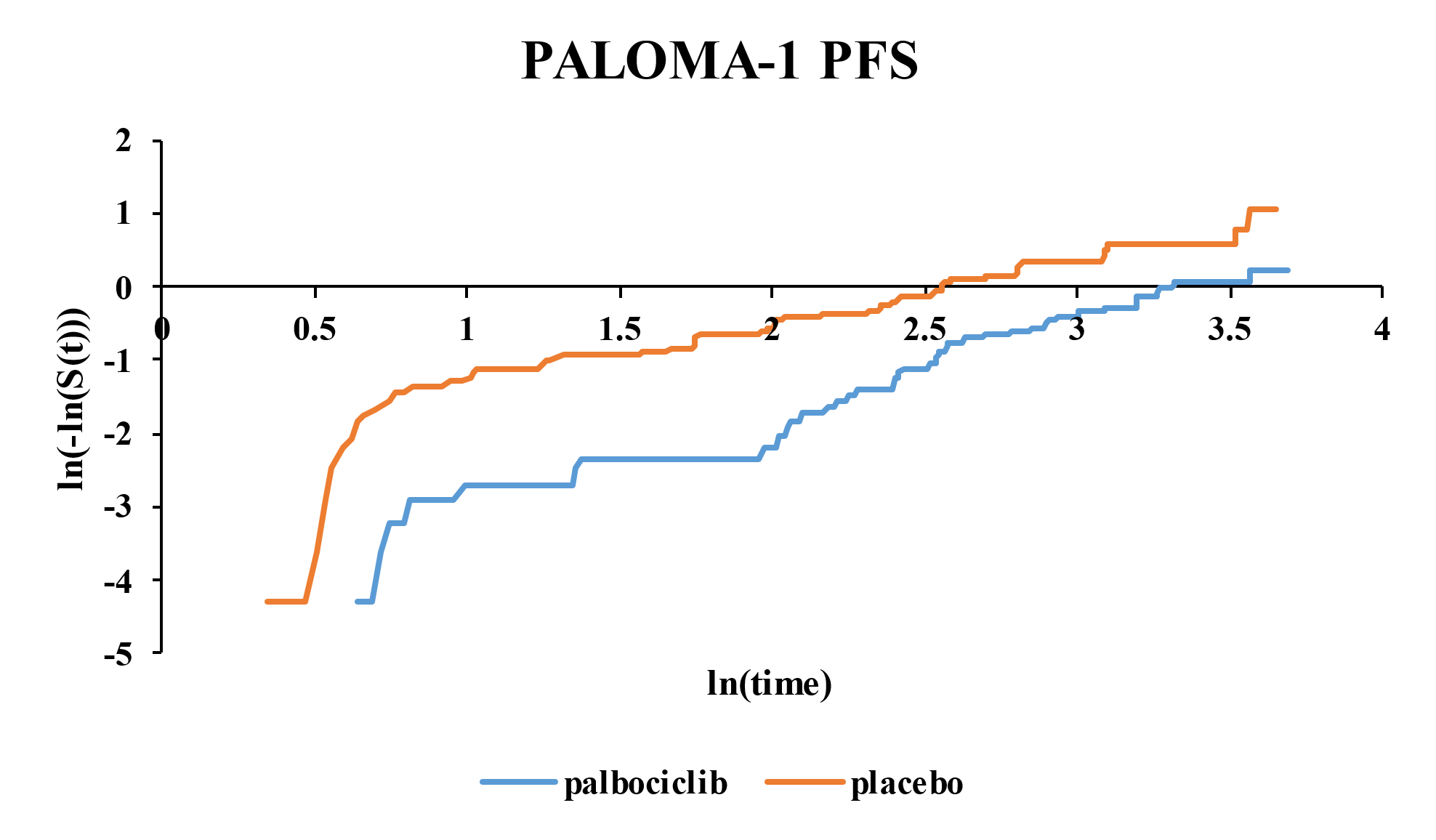 | 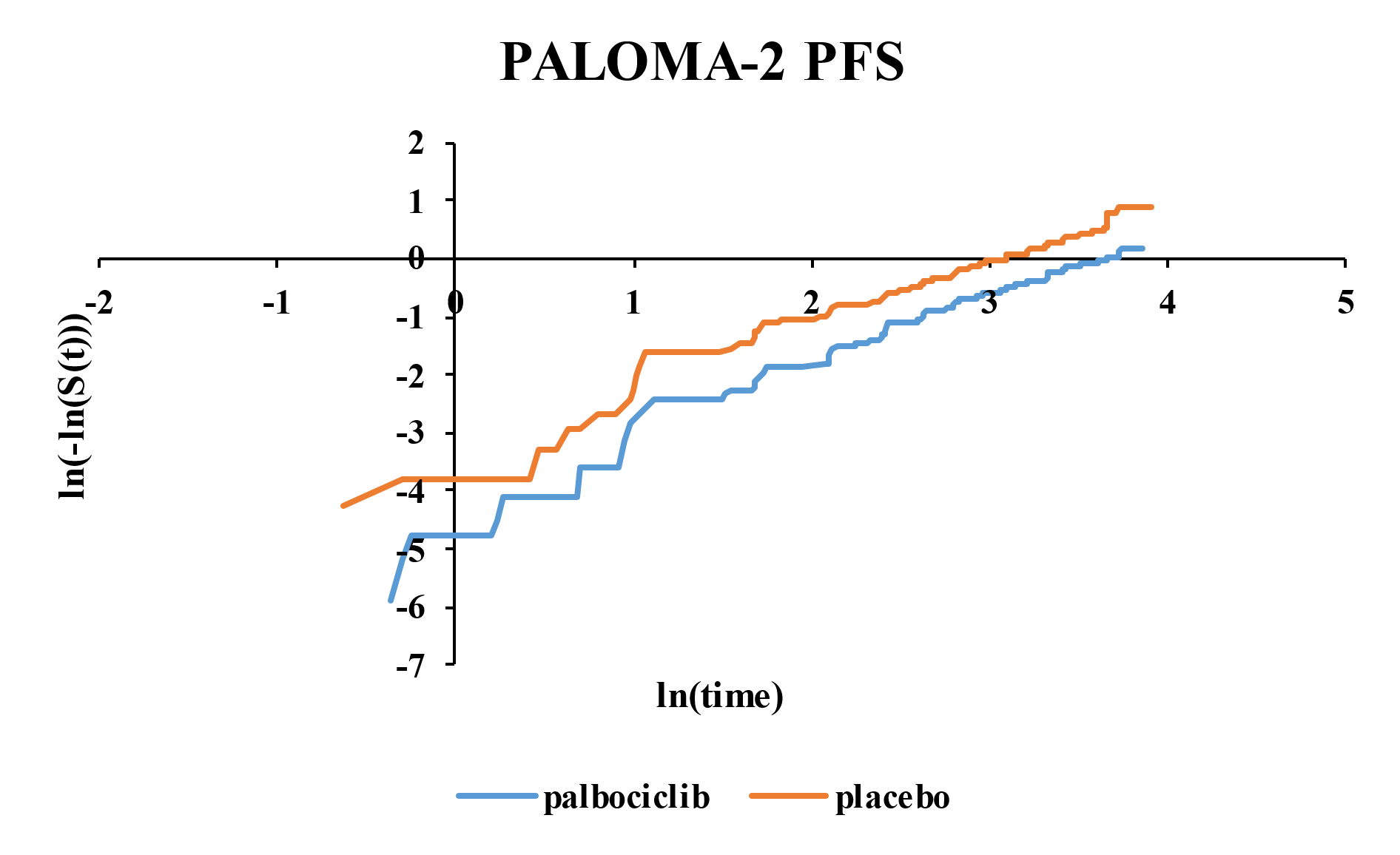 |
| 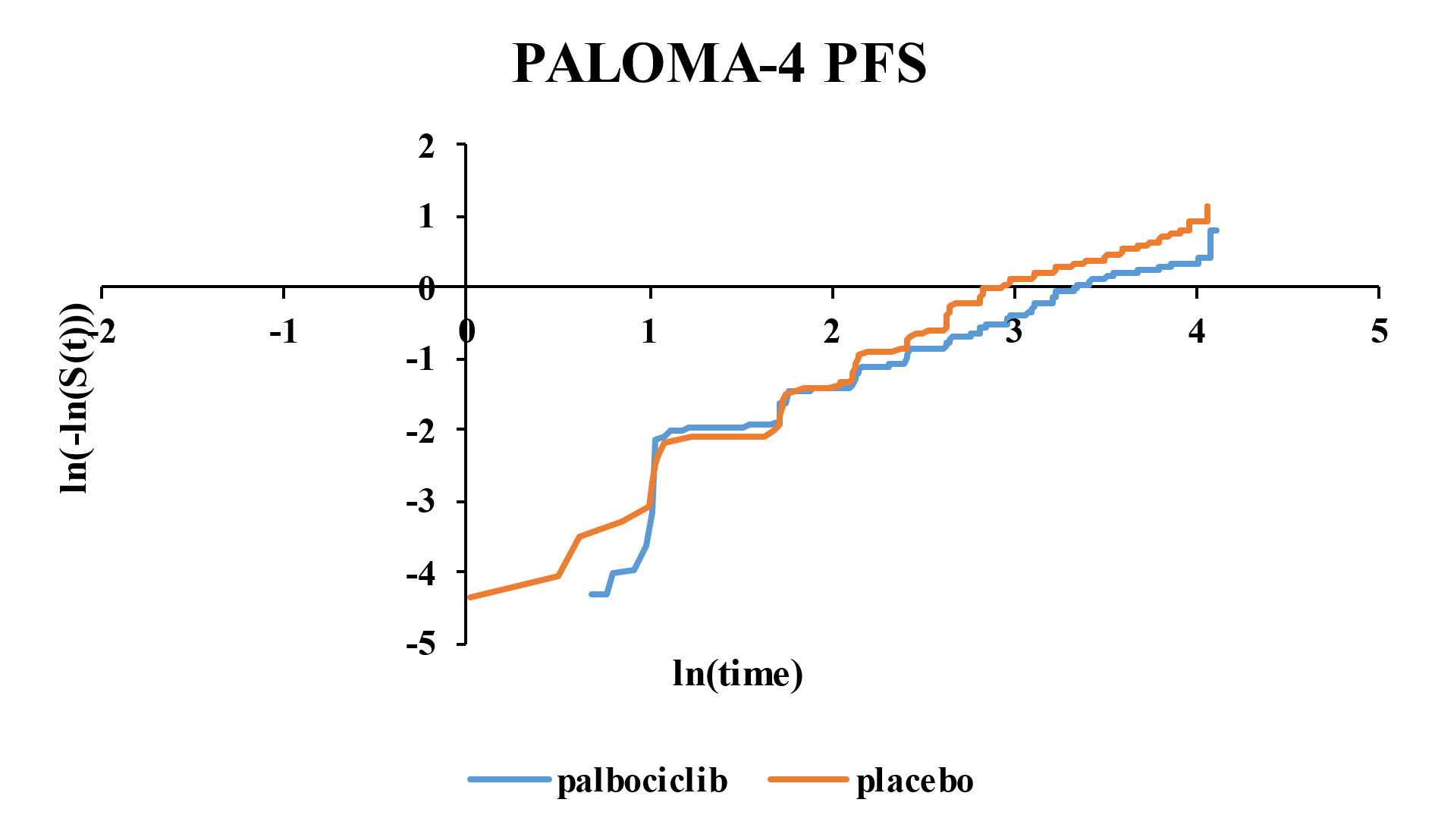 | 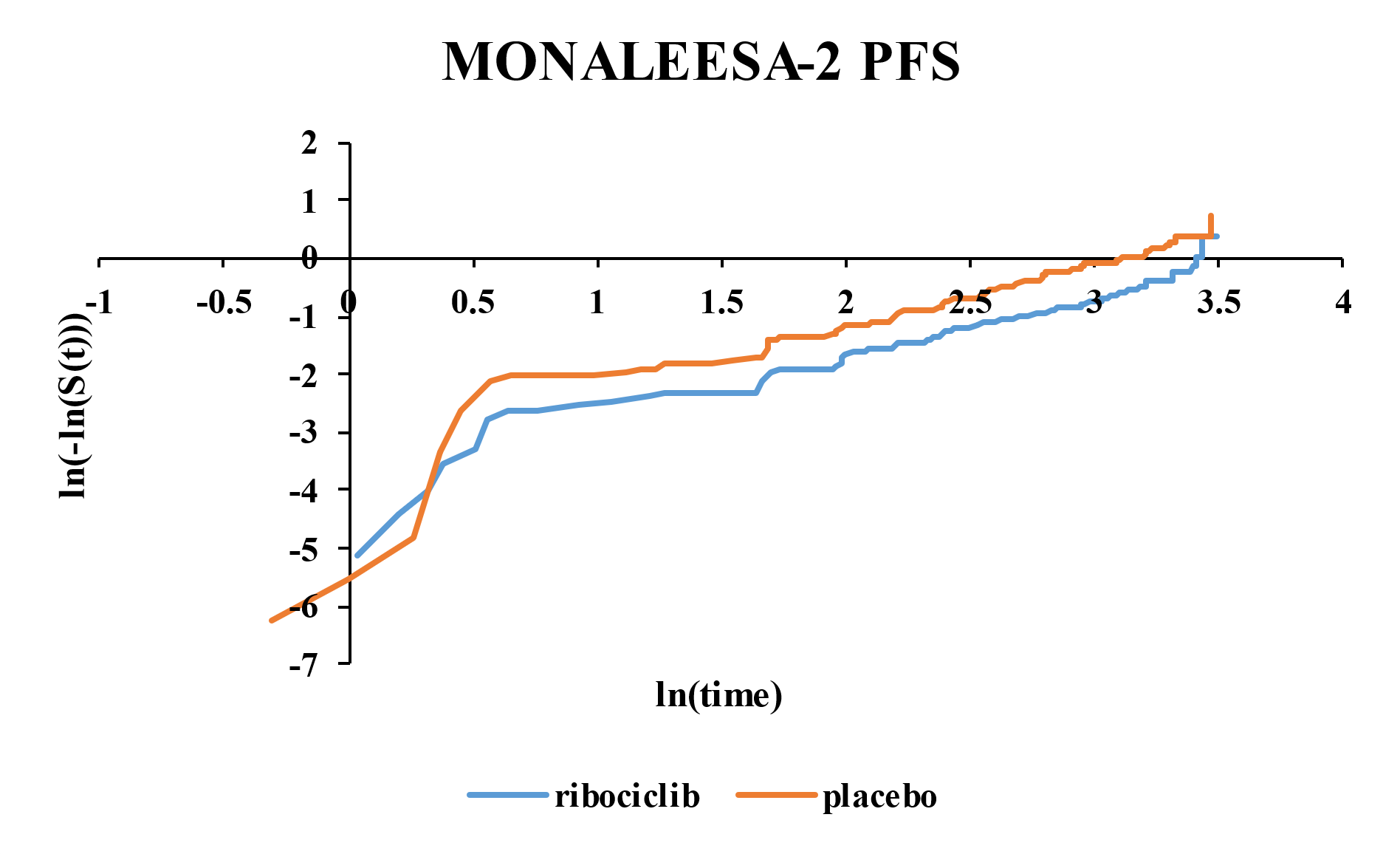 |

# Supplementary Figure S2. Risk of Bias Graph


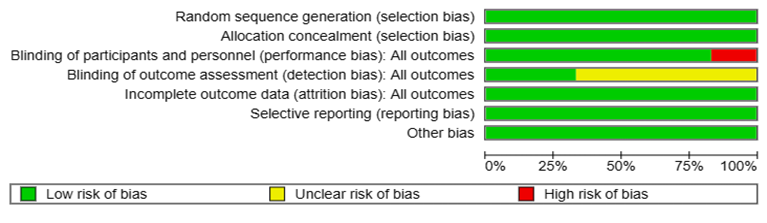


# Supplementary Figure S3. Risk of Bias Summary


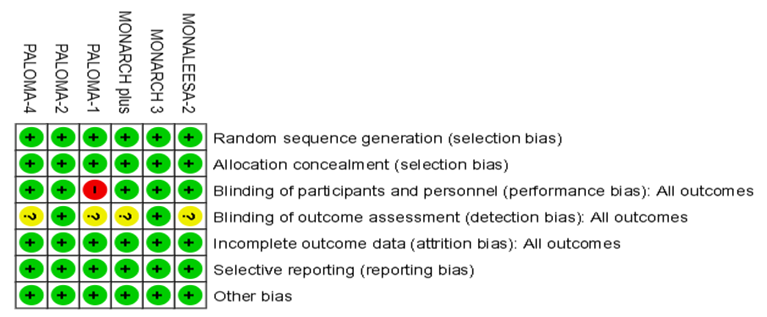


# Supplementary Table S3. AICs and BICs for Standard Parametric Models of Survival Curves for Anchor Treatment.

| **Distribution** | **PFS** | | **OS** | |
| --- | --- | --- | --- | --- |
|  | **AIC** | **BIC** | **AIC** | **BIC** |
| Standard Parametric Models | | | | |
| Exponential | 1143.974 | 1147.080 | 1236.002 | 1239.108 |
| Gamma | 1145.453 | 1151.665 | 1217.244 | 1223.456 |
| Gompertz | 1144.086 | 1150.297 | 1226.686 | 1232.898 |
| Weibull | 1145.955 | 1152.167 | 1218.400 | 1224.612 |
| Log-logistic | 1140.999 | 1147.211 | **1216.227** | **1222.439** |
| Log-normal | **1136.805** | **1143.017** | 1226.923 | 1233.135 |

# Supplementary Figure S4. Fitted Survival Curves of All Standard Models for Overall Survival, Progression Free Survival.

| **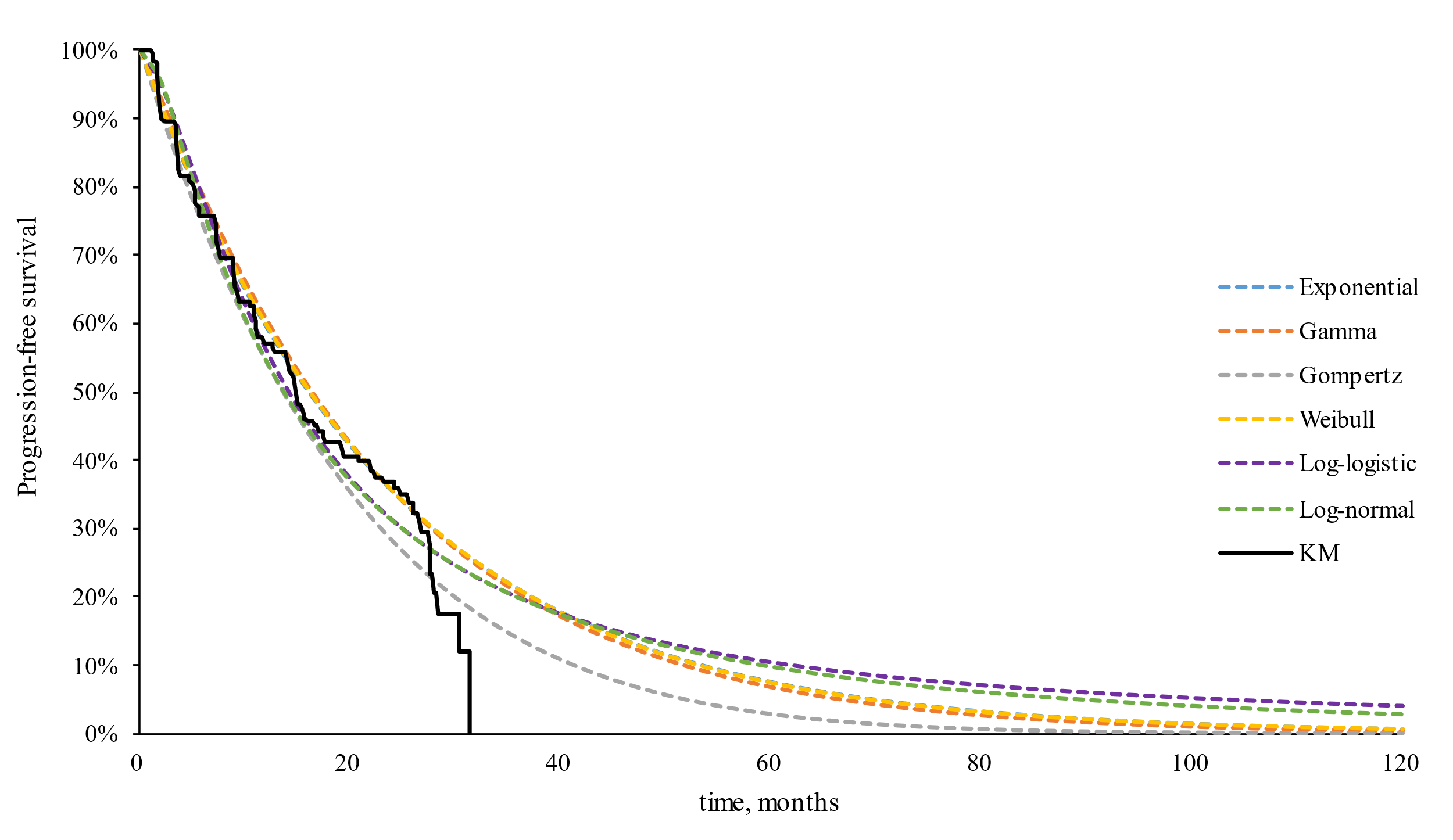** |
| --- |
| **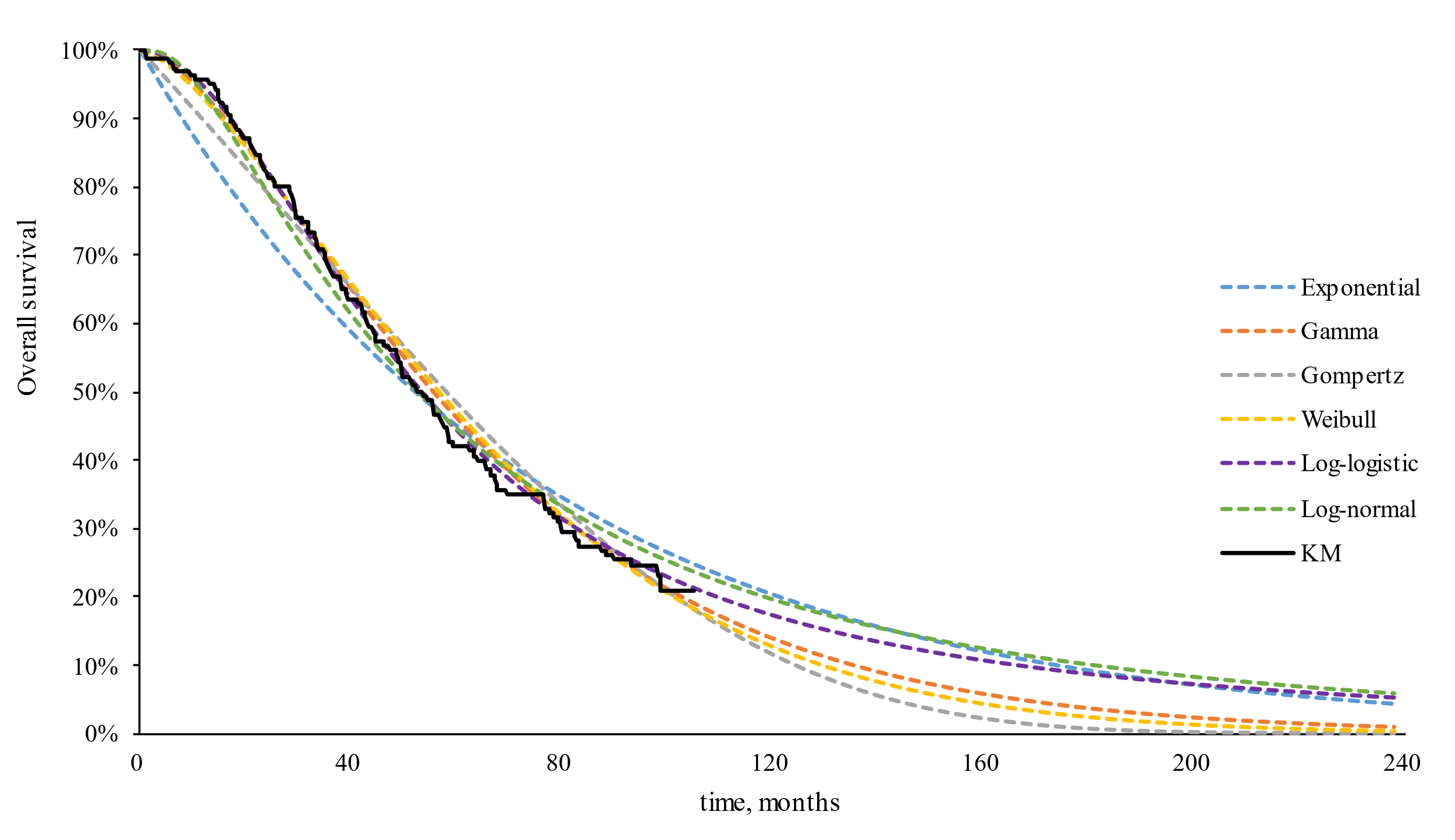** |

# Supplementary Figure S5. Forest plots for grade ≥ 3 AEs

| 1. **Neutropenia** | **(B) Leukopenia** |
| --- | --- |
| **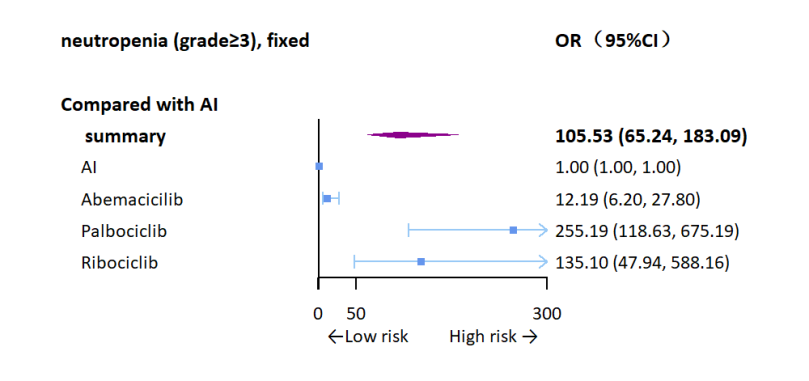** | **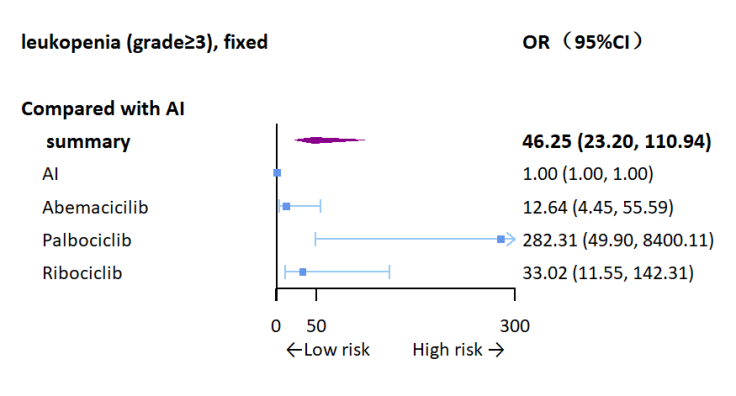** |
| **(C) Diarrhea** | **(D) Anemia** |
| **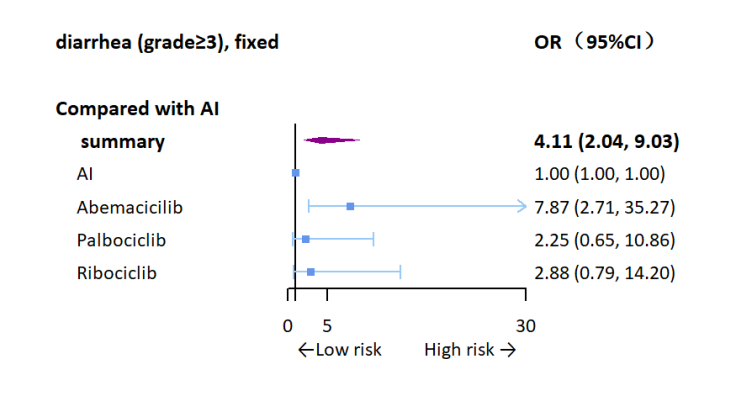** | **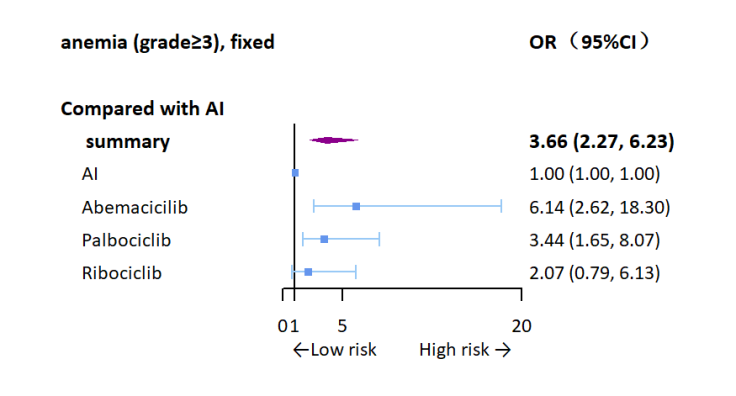** |
| **(E) ALT/AST increase** |  |
| **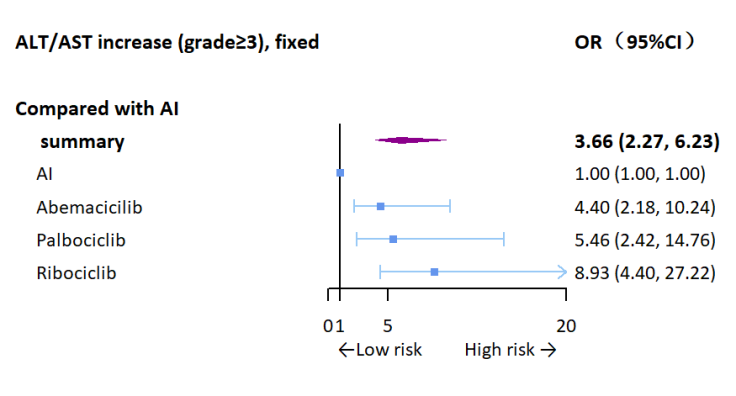** |  |
